# Social Trust, Literacy, and Lifelong Learning: A Comparison of the U.S. and Nordic Countries

**DOI:** 10.1093/geroni/igab046.2823

**Published:** 2021-12-17

**Authors:** Samuel Van Vleet, Phyllis Cummins, Abigail Helsinger

**Affiliations:** 1 Miami University, Oxford, Ohio, United States; 2 Scripps Gerontology Center, Miami University, Oxford, Ohio, United States

## Abstract

Societal social trust has been shown to be related to economic growth and equality. Low levels of social trust are especially consequential in aging societies and can result in low levels of social capital and greater inequality at older ages. Nordic countries are known for their greater social trust, access to education, economic productivity, and social equality. To better understand social trust promoters, we explored data from the 2012/2014 Program for the International Assessment of Adult Competencies (PIAAC) to examine relationships among social trust, basic skills (i.e., literacy), and non-formal education (NFE) participation for adults ages 45 to 65, in the U.S., Denmark, Finland, Norway, and Sweden. Additionally, through 19 key informant interviews and a review of the literature, we investigated the structure and availability of NFE across the five nations. As compared to the U.S., adults ages 45 - 65 in Nordic countries have higher levels of social trust (all Nordic countries; p < 0.001), lower rates of poor literacy skills (Finland, Norway, and Sweden; p <.001), greater rates of participation in NFE (Denmark and Sweden; p < 0.05). Through the availability of NFE, such as folk high schools and learning circles in Nordic countries, adults can participate in NFE at little or no cost. Similar programs are not available in the U.S. This research informs policy and practice for the provision of NFE, which is critical to increase levels of social trust, and in turn, to promote economic development, social equality and positive aging in the U.S.

